# Low‐Intensity Ultrasound‐Triggered Microbubble Cavitation Attenuates Diabetic Kidney Injury Partially by Reversing Endothelial Dysfunction

**DOI:** 10.1002/advs.77989

**Published:** 2026-10-06

**Authors:** Yubo Lai, Liang Zhang, Wenxin Tao, Jieyuan An, Lantian Wang, Zhaoyou Liu, Guodong Yang, Pengying Wu, Lijun Yuan

**Affiliations:** ^1^ Department of Ultrasound Medicine Tangdu Hospital Fourth Military Medical University Shaanxi China; ^2^ State Key Laboratory of Holistic Integrative Management of Gastrointestinal Cancers Department of Biochemistry and Molecular Biology Fourth Military Medical University Xi'an Shaanxi China

**Keywords:** cavitation, diabetic kidney disease, endothelial cell, mechanical modulation, microbubbles, therapeutic ultrasound

## Abstract

Herein, we developed a novel non‐invasive therapeutic strategy, namely ultrasound‐triggered cavitation of diagnostic‐grade microbubbles (MBs), which delivers favorable rhythmic mechanical stimuli to reverse this core pathological defect of diabetic kidney disease (DKD). By systematically optimizing parameters between low‐intensity pulsed ultrasound (LIPUS) and the clinically approved diagnostic MB contrast agent Sonazoid, we established a standardized protocol (Sonazoid: 0.008 µg per injection; LIPUS: 3.4‐4.1 MHz, mechanical index 0.2; 10 min per session, twice weekly for 4 weeks) to precisely deliver localized favorable rhythmic mechanical stimuli to the renal microvasculature. In the db/db mouse model of progressive DKD, this therapeutic intervention significantly improved renal function, reduced albuminuria, and mitigated glomerular and tubular histological lesions. Ultrasound localization microscopy (ULM) verified a remarkable enhancement in renal cortical microcirculatory perfusion. Transcriptomic profiling of renal tissues revealed down‐regulation of key pathways involved in endothelial dysfunction, leukocyte chemotaxis, and fibrosis, accompanied by a shift toward an anti‐inflammatory and anti‐fibrotic phenotypic state. Our findings demonstrate that this therapeutic strategy generates favorable mechanical modulation at the microvascular level, effectively retarding DKD progression by restoring microvascular homeostasis and suppressing pathogenic inflammatory and fibrotic cascades. This approach offers a promising and translational platform technology for the management of microvascular diseases beyond DKD.

## Graphical abstract

Non‐invasive low‐intensity pulsed ultrasound ‐triggered cavitation of clinically approved diagnostic‐grade microbubbles delivers favorable mechanical stimuli to renal microvasculature. This intervention restores renal microvascular homeostasis, alleviates DKD‐associated histological and functional damage, and suppresses inflammatory and fibrotic cascades.

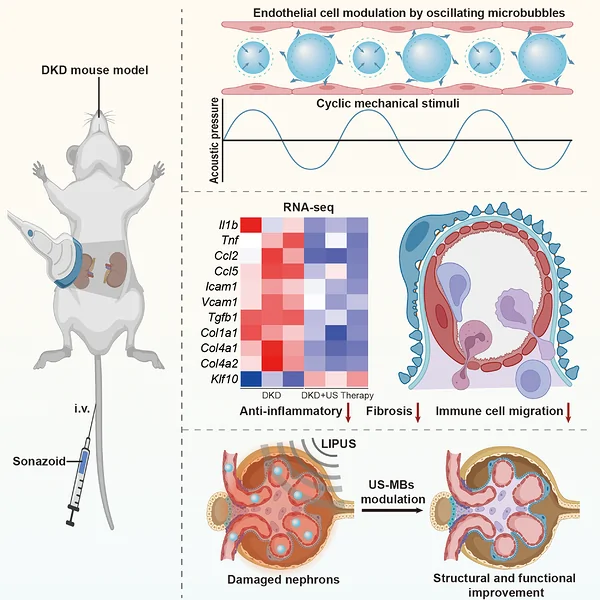

## 1 Introduction

DKD is one of the most severe microvascular complications of diabetes mellitus and represents the leading cause of chronic kidney disease worldwide [1, 2]. Approximately 40% of individuals with diabetes mellitus will progress to DKD [3, 4], and its incidence continues to rise even as the incidence of other diabetic complications declines [4, 5]. This growing burden poses a significant threat to global public health and highlights the need for new therapeutic strategies.

Endothelial cell (EC) dysfunction has emerged as a key pathological mechanism driving the progression of DKD [6, 7]. Under diabetic conditions, the combined effects of hyperglycemia, advanced glycation end products (AGEs), and reactive oxygen species (ROS) create a hostile microenvironment that impairs glomerular endothelial function. As essential components of the renal filtration barrier, ECs regulate substance exchange, coagulation, and inflammatory responses under normal physiological conditions. In DKD, however, this pathological environment damages the endothelial glycocalyx, increases vascular permeability, and triggers pro‐inflammatory signaling cascades that worsen renal injury [8]. Restoring endothelial homeostasis has therefore emerged as a promising therapeutic target for treating DKD.

Ultrasound (US) is a non‐invasive technique that can penetrate biological tissues to reach deep organs, with excellent spatiotemporal resolution. Beyond its diagnostic utility, growing evidence supports its therapeutic potential for various diseases [9]. The interaction between US and MBs has shown favorable clinical safety [10, 11]. At low acoustic pressures, US induces cavitation, which is characterized by the cyclic compression and expansion of MBs [12, 13]. This oscillation exerts cyclic mechanical stimulation on the vascular endothelium [14], mimicking the physiological hemodynamic forces that regulate normal vascular function. Vascular ECs are naturally mechanosensitive [7, 15], and their gene expression profiles and functional homeostasis are closely regulated by hemodynamic shear forces [16]. This mechanotransduction ability provides a theoretical basis for using US‐triggered cavitation to deliver beneficial mechanical stimuli to dysfunctional endothelium. Harnessing this physical principle could potentially restore endothelial function and interrupt the pathological cascade that drives DKD progression.

In this study, we employed widely used clinically LIPUS in conjunction with Sonazoid, a clinically approved and highly stable diagnostic MB contrast agent. Through systematic optimization of the key interaction parameters, we established a protocol for US‐mediated mechanical modulation via diagnostic‐grade MBs cavitation, hereafter denoted as US therapy. This strategy delivers controlled cyclic mechanical stimulation to the renal microvasculature. We validated its therapeutic efficacy in a db/db mouse model of progressive DKD and preliminarily elucidated the underlying mechanisms.

## 2 Result

### 2.1 Establishment of the US Therapy Protocol

Under the pathological condition of DKD, ECs exhibit upregulated expression of genes involved in apoptosis and inflammation‐related signaling pathways [17]. To identify optimal therapeutic parameters, we first performed in vitro transcriptomic analysis on human umbilical vein endothelial cells (HUVECs) cultured under high glucose (HG) conditions to model diabetic endothelial dysfunction. Cells were subjected to US treatment at a fixed frequency (3.4–4.1 MHz) with graded mechanical index (MI) values (Figure 1A). The Cell Counting Kit‐8 (CCK‐8) assay was applied to evaluate cell viability following different interventions. The results demonstrated that cell viability was significantly decreased in the HG‐injured model compared with the control group. Notably, interventions with MI 0.2, 0.3, and 0.4 markedly restored cell viability relative to the HG group (*P* < 0.05), whereas no significant difference was observed between the MI = 0.5 group and the HG group (Figure 1B).

**FIGURE 1:**
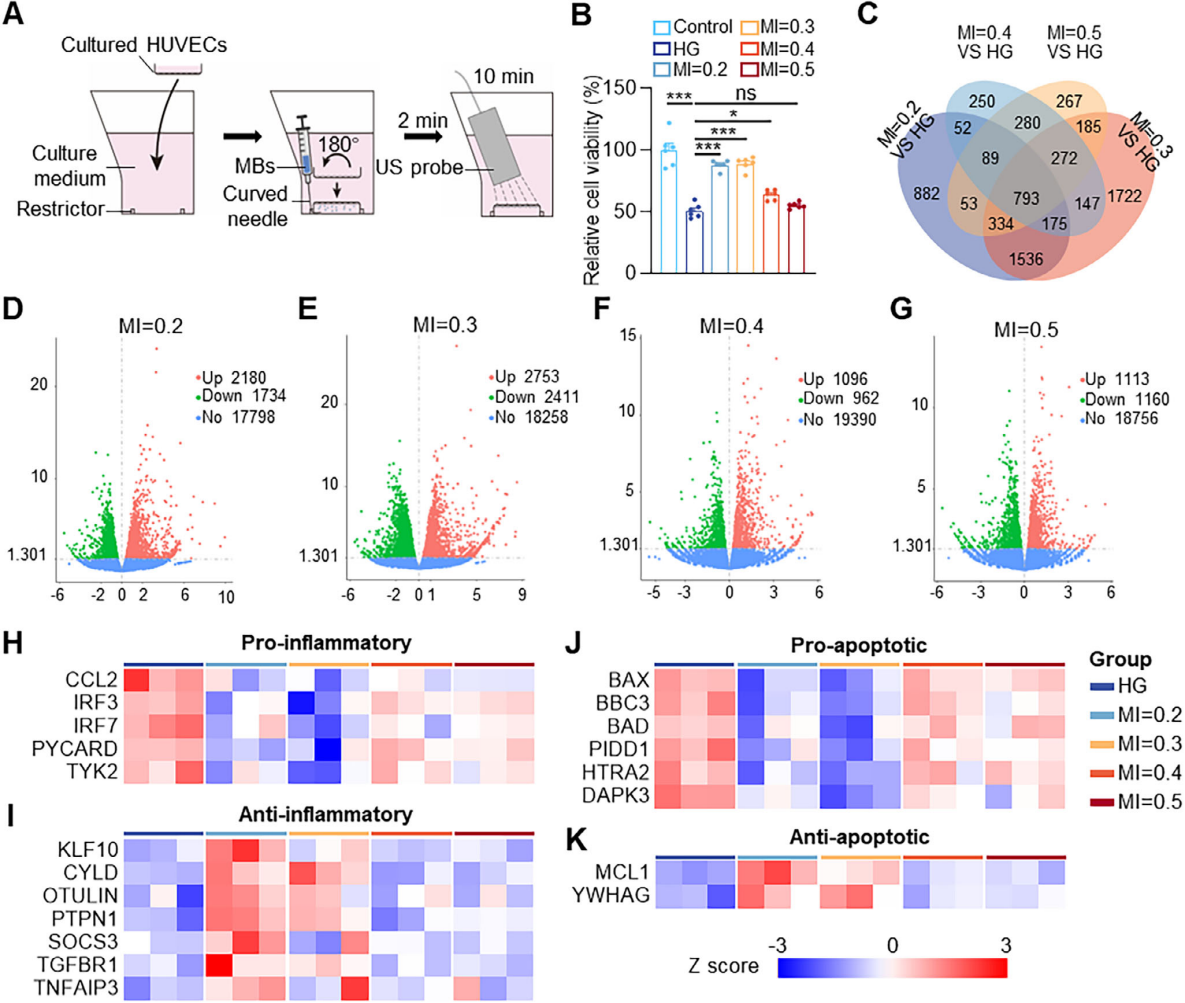
Optimization of therapeutic parameters modulates endothelial gene expression profiles in vitro. (A) Schematic of the in vitro US + MBs intervention system. (B) Cell viability was evaluated using the CCK‐8 assay, with all values normalized to the control group. n = 6. (C) Venn diagram of DEGs from transcriptome sequencing. (D–G) Volcano plots of DEGs in HUVECs following treatment with US‐MBs at MI values of 0.2, 0.3, 0.4, and 0.5, compared with the high glucose group. (H) Heatmap of z‐score normalized expression of pro‐inflammatory genes. (I) Heatmap of z‐score normalized expression of anti‐inflammatory genes. (J) Heatmap of z‐score normalized expression of pro‐apoptotic genes. (K) Heatmap of z‐score normalized expression of anti‐apoptotic genes. HUVECs: human umbilical vein endothelial cells; HG: high glucose; MI: mechanical index. n.s., not significant; ^*^ *P* <0.05, ^***^ *P* < 0.001.

Transcriptome sequencing revealed significant alterations in gene expression profiles following treatment (Figure 1C–G). The MI 0.2 group yielded the highest number of differentially expressed genes (DEGs) (Figure 1D), characterized by the most pronounced downregulation of pro‐inflammatory (Figure 1H) and pro‐apoptotic (Figure 1J) genes, alongside upregulation of anti‐inflammatory (Figure 1I) and anti‐apoptotic genes (Figure 1K). These beneficial transcriptional shifts were absent at higher MI values (Figure 1H–K). Additional sequencing analysis results are available in Figures S1–S3.

We next screened parameters in vivo using db/db DKD model mice [18] (Figure 2A). To establish an efficient in vivo screening system, we prioritized inflammatory markers over renal functional parameters as the primary screening endpoint, as inflammation acts as an earlier and more sensitive indicator of therapeutic efficacy [3, 19]. We retained MI values of 0.2 and 0.3, which exhibited superior anti‐inflammatory and anti‐apoptotic effects in our in vitro experiments, and further screened the optimal LIPUS frequency and MI suitable for in vivo intervention. The results showed that US at 3.4–4.1 MHz with MI = 0.2 produced the most prominent effects in alleviating renal macrophage infiltration (Figure 2B,C) and downregulating the gene expression of pro‐inflammatory cytokines, including *Il6*, *Il8*, *Tnfa*, *Tgfb1*, and *Ccl2* (Figure 2D–I). Subsequent MBs dose screening experiments identified 0.008 µg Sonazoid per mouse as the optimal dose for alleviating renal inflammation (Figure S4). We further verified the biosafety of US + MBs intervention through H&E staining and F4/80‐based macrophage immunohistochemistry of major visceral organs (Figures S5 and S6), together with serum detection of pro‐inflammatory cytokines and liver function parameters (Figure S7). Accordingly, the optimal intervention protocol for US therapy was established: 0.008 µg Sonazoid per mouse, combined with LIPUS stimulation at 3.4–4.1 MHz and an MI of 0.2. We further confirmed that the optimized US insonation field could cover both kidneys, with substantial microbubble retention maintained in the renal parenchyma within 10 min following treatment under these parameters (Figure S8).

**FIGURE 2:**
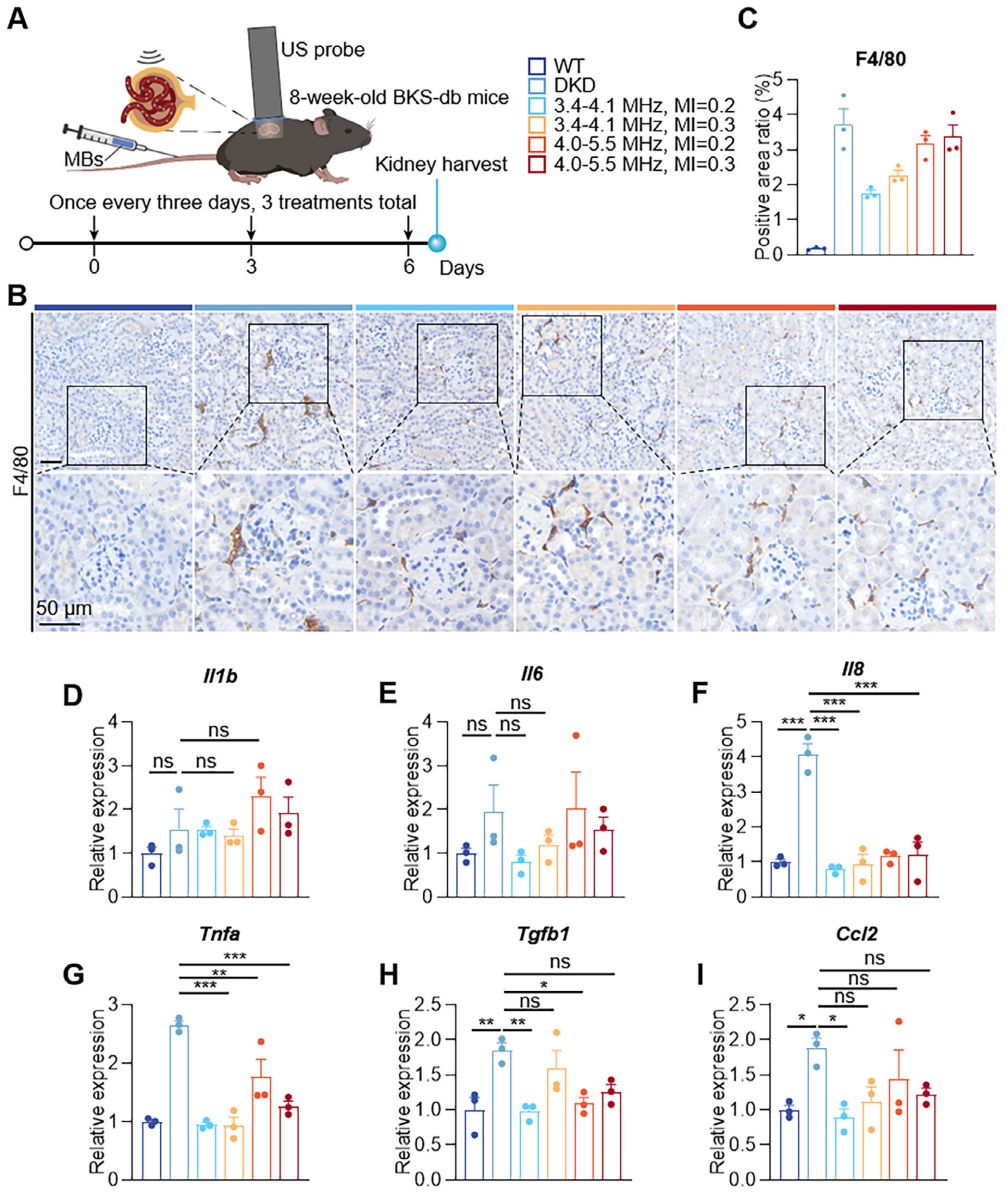
In vivo screening identifies the optimal therapeutic protocol. (A) Schematic of the in vivo parameter screening workflow. (B) Representative immunohistochemical staining for the macrophage marker F4/80. (C) Quantitative analysis of F4/80‐positive area. (D‐I) Relative mRNA expression of pro‐inflammatory and pro‐fibrotic genes (*Il1b, Il6, Il8, Tnfa, Tgfb1, Ccl2*) in renal tissues during screening. Data are mean ± SEM (n = 3); one‐way ANOVA; n.s., not significant; ^*^ *P* <0.05, ^**^ *P* < 0.01, ^***^ *P* < 0.001.

### 2.2 US Therapy Protocol Improves Renal Function and Structure

Using the established protocol (twice weekly for 4 weeks; Figure 3A), we assessed therapeutic efficacy in db/db mice. US therapy did not affect body weight or fasting blood glucose (Figure S9B, C). However, it significantly improved renal function, as evidenced by reduced serum urea (Figure S9D) and serum uric acid (Figure S9F). Although a numerical decrease in serum creatinine was observed, the difference did not reach statistical significance (Figure S9E). This intervention significantly improved renal function at the end of treatment, with reduced urine albumin‐to‐creatinine ratio (UACR) and elevated GFR in the treatment group versus the DKD group, and these renoprotective effects persisted for at least 2 weeks (Figure 3B,C). However, UACR rebounded more rapidly in the treatment group over the 2‐week post‐treatment follow‐up period (Figure 3B), suggesting that sustained intervention is required to preserve renoprotective benefits given the persistent underlying diabetic risk factors. In addition, we confirmed that US‐only intervention for a 4‐week period failed to alleviate renal inflammation or restore renal function in DKD mice (Figure S10).

**FIGURE 3:**
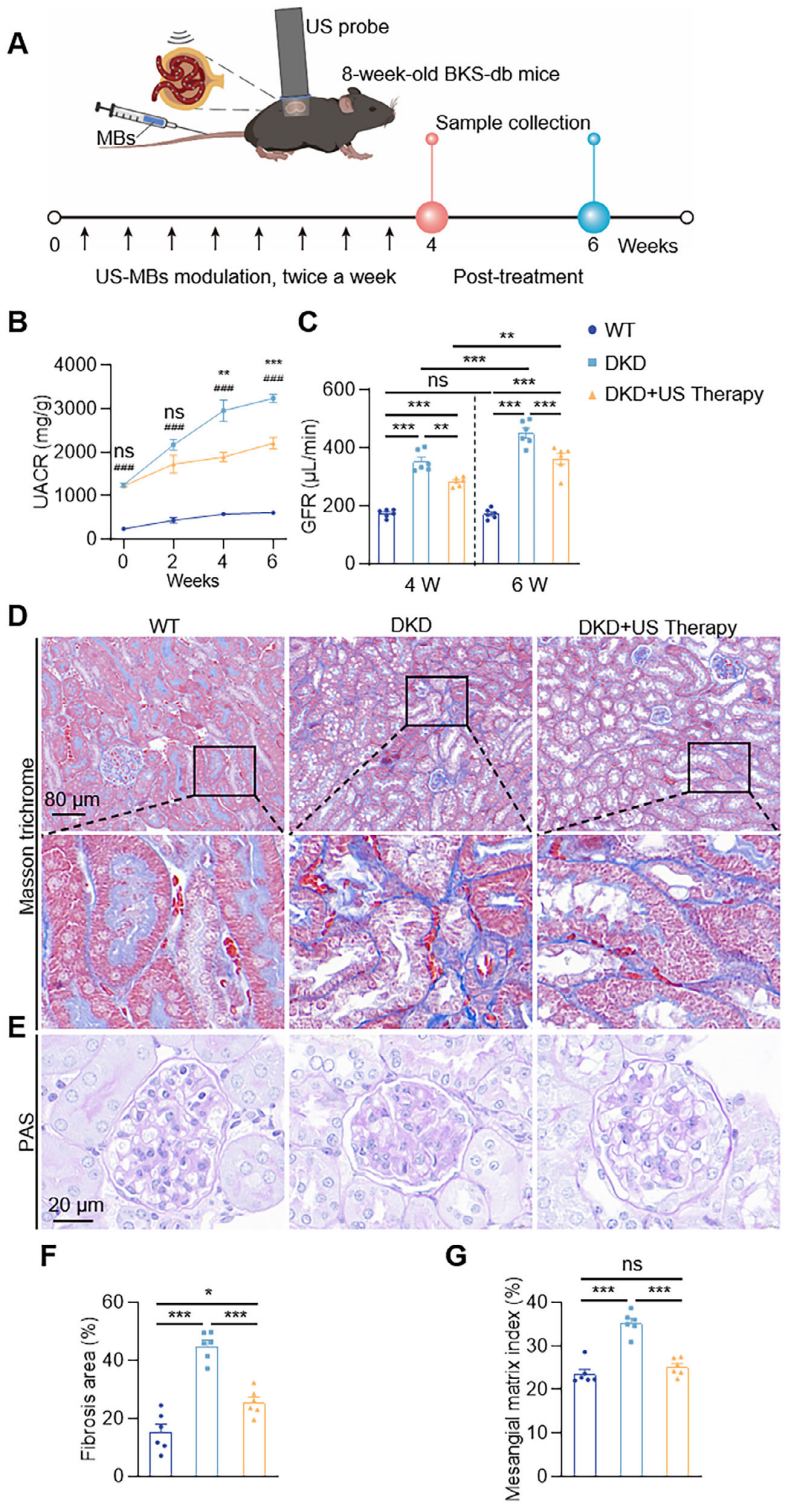
US therapy ameliorates renal function and structural integrity in DKD mice. (A) Schematic diagram of the 4‐week in vivo therapeutic regimen and the time points of sample harvesting. (B) Urine albumin‐to‐creatinine ratio (UACR) measured during the US therapy period and at 2 weeks after treatment cessation. ^**^ *p* < 0.01, ^***^ *p* < 0.001 for DKD + Therapy vs. DKD; ###*p* < 0.001 for DKD + Therapy vs. WT. (C) Glomerular filtration rate (GFR) determined at the end of the US therapy course and at 2 weeks post‐intervention. (D) Representative micrographs of Masson's trichrome staining for renal fibrosis assessment (collagen fibers stained blue). (E) Representative micrographs of periodic acid‐Schiff (PAS)‐stained glomerular sections. (F) Quantitative analysis of the relative renal interstitial fibrosis area.(G) Quantitative analysis of the glomerular mesangial matrix area. Data are mean ± SEM (n = 6); one‐way ANOVA; n.s., not significant; ^*^ *P* <0.05, ^**^ *P* <0.01, ^***^ *P* <0.001.

Histopathological analysis correlated these functional improvements with structural preservation. Treated mice exhibited reduced renal fibrosis (Figure 3D,F) and ameliorated mesangial matrix expansion (Figure 3E,G). These structural protective effects were likewise sustained for at least 2 weeks after treatment completion (Figure 3F,G). Transmission electron microscopy further confirmed protection of the glomerular filtration barrier, demonstrating a thinner glomerular basement membrane (GBM, Figure S9G,H) and narrower podocyte foot process width (Figure S9G,I) in the treatment group. Concurrently, mice with US therapy showed a significant increase in podocyte density, a parameter typically lost in DKD progression, as quantified by NPHS2 [20] immunofluorescence staining (Figure S11).

### 2.3 US Therapy Enhances Renal Microcirculatory Perfusion

ULM revealed significantly improved renal cortical perfusion following US therapy (Figure 4A). Quantitative analysis showed increased vessel ratio (Figure 4B), mean vascular density (Figure 4C), mean microcirculatory flow velocity (Figure 4D), and perfusion index (Figure 4E) in treated mice, indicating alleviation of microcirculatory dysfunction (Figure S12).

**FIGURE 4:**
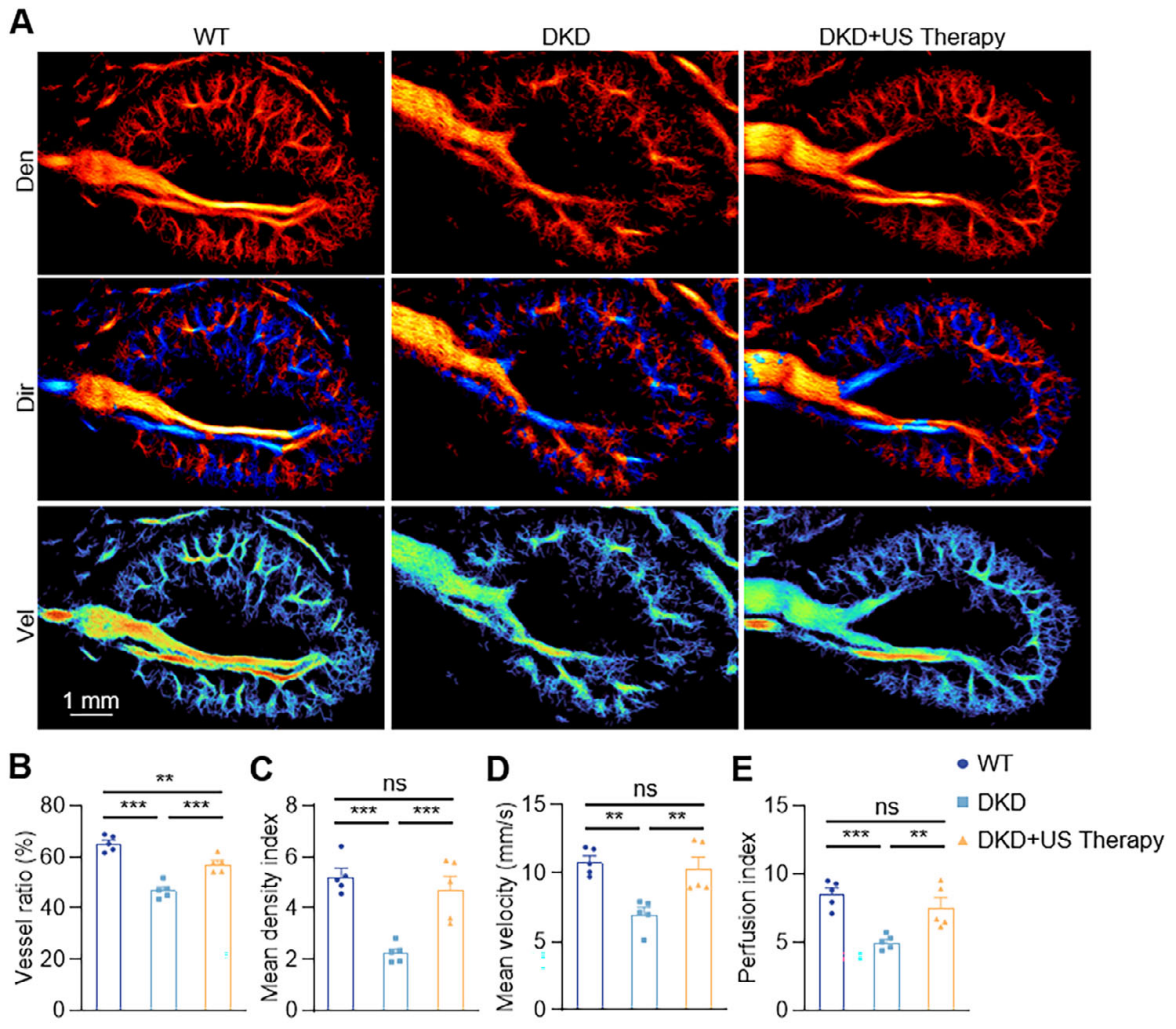
US therapy enhances renal microcirculatory perfusion in DKD mice. (A) Representative ultrasound localization microscopy images of renal cortical microvasculature. (B) Microvessel ratio. (C) Mean microvessel density. (D) Mean microcirculatory flow velocity. (E) Perfusion index. Data are mean ± SEM (n = 5); one‐way ANOVA; n.s., not significant; ^**^ *p* < 0.01, ^***^ *p* < 0.001.

### 2.4 Mechanistic Insights From Transcriptomic Profiling

Transcriptomic analysis of renal tissues elucidated potential mechanisms. GO enrichment of DEGs highlighted pathways related to endothelial function, oxidative stress, and leukocyte‐mediated inflammation (Figure 5A and Figure S13). Gene set enrichment analysis (GSEA) using disease ontology gene sets showed a downward trend for chronic kidney failure signatures (Figure 5B).

**FIGURE 5:**
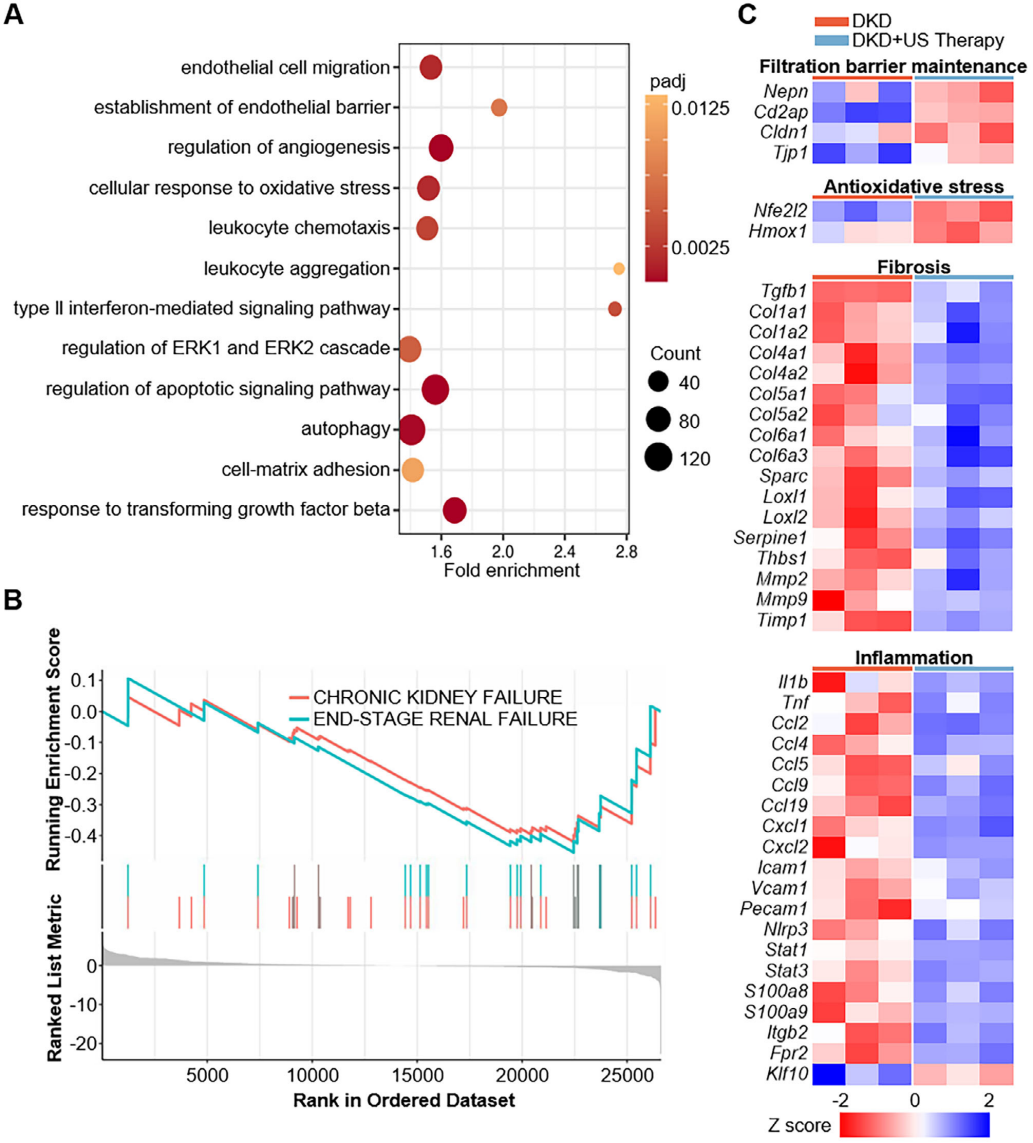
Transcriptomic analysis reveals restoration of microvascular homeostasis and suppression of pathogenic inflammatory/fibrotic cascades. (A) GO enrichment analysis of renal tissue DEGs, highlighting pathways related to endothelial function, fibrosis, and leukocyte‐mediated inflammation. (B) GSEA plots showing suppression of disease‐associated signatures (“CHRONIC KIDNEY FAILURE” and “END‐STAGE RENAL FAILURE” gene sets). (C) Z‐score heatmap of key genes involved in filtration barrier maintenance, antioxidant response, fibrosis, and inflammation, demonstrating a shift toward an anti‐inflammatory and anti‐fibrotic phenotype.

A DEG heatmap demonstrated upregulation of genes involved in filtration barrier maintenance (e.g., *Nepn*) and antioxidant response (e.g., *Nfe2l2, Hmox1*). Concurrently, pro‐fibrotic (*Tgfb1, Col4a1, Col4a2*) and pro‐inflammatory (*Il1b, Tnf, Ccl2, Ccl5, Icam1*) genes were downregulated, while the anti‐inflammatory gene Klf10 was upregulated (Figure 5C). These data confirm that the therapy induces an anti‐inflammatory and anti‐fibrotic transcriptomic shift, potentially mediated through improved endothelial function and suppression of leukocyte chemotaxis and adhesion.

## 3 Discussion

This study introduces a novel non‐invasive therapeutic strategy, US‐triggered cavitation of diagnostic‐grade MBs, for the treatment of DKD. By systematically optimizing the combination parameters of LIPUS and the clinically approved MB agent Sonazoid, we established an MB cavitation based therapeutic protocol that delivers localized favorable rhythmic mechanical stimuli to the renal microvasculature. In a db/db mouse model of progressive DKD, this intervention significantly improved renal function, reduced albuminuria, attenuated glomerular and tubular histological damage, and enhanced renal cortical microcirculatory perfusion as verified by ULM. Transcriptomic profiling revealed a shift toward an anti‐inflammatory and anti‐fibrotic phenotypic state, with downregulation of key pathways involved in endothelial dysfunction, leukocyte chemotaxis, and fibrosis.

Our findings align with and extend the growing literature on the therapeutic application of US‐mediated mechanical stimuli [9]. Distinct from High‐intensity focused US that induces irreversible tissue ablation and sonogenetics that relies on genetically modified ion channels for signal activation, our approach exploits low‐acoustic‐pressure LIPUS to mediate mild, rhythmic mechanical interaction with circulating Sonazoid MBs, thereby triggering cavitation. This biomechanical paradigm is consistent with emerging evidence that physiological mechanical force is indispensable for maintaining tissue homeostasis. ECs are highly mechanosensitive cells, and laminar shear stress‐derived mechanical cues are well recognized to sustain vascular quiescence and barrier integrity [7]. In the diabetic microenvironment, disrupted hemodynamics lead to the loss of endogenous protective mechanical signals, which initiates early vascular lesions [7]. Mechanistically, cyclic mechanical stimulation generated by MB cavitation mimics physiological hemodynamic stimulation, exerting favorable mechanical modulation on renal ECs. Such exogenous mechanical intervention restores endothelial mechano‐homeostasis and reverses the depletion of protective mechanical cues in damaged renal microvessels, laying a foundational mechanical basis for subsequent anti‐injury effects.

EC dysfunction acts as the central hub and core pathological driver of DKD progression, forming a closed pathological cascade of endothelial injury‐inflammation‐fibrosis throughout disease development [6, 21]. Persistent hyperglycemia and metabolic disturbance first impair the integrity of glomerular and peritubular endothelium, triggering endothelial apoptosis, increased vascular permeability, and aberrant secretion of adhesion molecules and pro‐inflammatory cytokines. Dysfunctional ECs further recruit circulating leukocytes and monocytes for renal infiltration, amplify local chronic inflammation and immune activation, induce aberrant coagulation and elevated microcirculatory pressure, and drive fibroblast activation. These cascading events ultimately aggravate extracellular matrix accumulation, leading to irreversible glomerular and interstitial fibrosis [22, 23]. In this pathological axis, the US therapeutic strategy described herein directly targets injured endothelial barriers. It ameliorates renal microcirculation and simultaneously downregulates the endothelial activation marker *Vcam1* and restrains excessive endothelial activation. Meanwhile, this intervention significantly suppresses pro‐inflammatory genes (*Il1b, Tnf, Ccl2, Ccl5, Icam1*) and pro‐fibrotic genes (*Tgfb1, Col4a1, Col4a2*), while elevating the expression of antioxidant stress‐related genes (*Nfe2l2, Hmox1*) and anti‐inflammatory genes such as *Klf10*. Transcriptomic results collectively reveal the multi‐stage protective mechanism of the US therapy: it directly repairs damaged endothelial barriers and limits endothelial overactivation; secondarily inhibits inflammatory cell adhesion and chemotaxis by reducing endothelial pro‐inflammatory mediators; and blocks inflammation‐mediated fibrotic responses via transcriptional repression of pro‐fibrotic signaling pathways.

However, our transcriptomic analyses primarily reflect downstream phenotypic and transcriptional changes induced by mechanical stimulation. The direct mechanosensing molecules and upstream signaling cascades responsible for perceiving the US therapy remain poorly elucidated. Accumulated evidence has demonstrated that cells rely on multiple mechanosensors to perceive mechanical stimuli, including ion channels such as the Piezo, TRP, K2P, and MscL families, as well as membrane proteins represented by prestin and integrin–cytoskeleton complexes [9, 24]. These structures convert extracellular physical cues into intracellular biochemical signals. Downstream mechanosensitive transcription factors, such as KLF2/4 and YAP/TAZ, further govern endothelial inflammation, proliferation, and fibrosis [16, 25]. We speculate that rhythmic mechanical force generated by US‐triggered MB cavitation is initially captured by endothelial membrane mechanoreceptors, which initiates intracellular mechanotransduction, remodels cytoskeleton organization, and ultimately inhibits the NF‐κB and TGF‐β/Smad pro‐inflammatory and pro‐fibrotic cascades. Although our transcriptomic data delineate downstream differential gene expression, further targeted studies are required to identify core mechanosensitive targets and clarify the temporal order of mechanical signal transduction in renal ECs.

From a broader translational perspective, the therapeutic advantages of this microvascular mechanical therapy are not limited to DKD. Endothelial dysfunction is a common hallmark of numerous microcirculation‐related disorders [26, 27], other diabetic complications [28], metabolic [29], cardiovascular [30], neurological [31], and dermatological diseases [32]. The non‐invasive, focal, and tunable nature of US‐triggered cavitation presents a promising and translatable platform technology. By adjusting parameters such as frequency, mechanical index, and MB dosage, the therapy could potentially be adapted to different vascular beds and disease states characterized by impaired perfusion, chronic inflammation, or fibrotic remodeling.

In the present study, non‐focused US was applied directly to the abdominal surface, exposing both kidneys simultaneously. While this approach ensures uniform bilateral renal treatment, it also unavoidably subjects surrounding tissues, including portions of the liver, spleen, intestine, and intra‐abdominal blood vessels, to the same cyclic mechanical forces. We performed preliminary histological safety assessments using H&E staining and F4/80 immunohistochemistry in the kidney, liver, and spleen, and observed no overt signs of tissue injury or heightened inflammation, supporting an acceptable safety profile for this treatment regimen. It should be acknowledged that real‐time cavitation activity in these off‐target organs and within the vascular compartment was not directly monitored in the current study. Dedicated cavitation detection and mapping techniques will be required in future investigations to quantitatively delineate the extent and biological consequences of cavitation events in non‐targeted tissues. Beyond these spatial considerations, the safety of repeated MB administration represents an equally critical factor for long‐term clinical deployment. Sonazoid, a clinically approved US contrast agent formulated with lipid‐shelled perfluorobutane MBs, exhibits a well‐validated safety profile with inherently minimal immunogenicity, owing to its biocompatible phospholipid envelope and biologically inert perfluorocarbon core. Large‐scale clinical practice and post‐marketing surveillance have confirmed that adverse events associated with Sonazoid are typically mild and self‐limiting, and no severe cumulative immune toxicity has been documented with repeated dosing [33, 34]. In the present study, our histopathological data consistently support the favorable long‐term biosafety of repeated US therapy.

Several limitations of this study warrant consideration and highlight directions for future research. First, while we identified an optimal parameter set, the specific cavitation parameters remain unclear; dedicated cavitation detection techniques are required in future studies to delineate the precise cavitation dynamics and their respective contributions to renal protection. Second, the therapeutic window for US parameters and MB dose remains to be fully defined. Third, the precise molecular sensors and signaling cascades that transduce the acoustic rhythmic mechanical stimulus into the observed anti‐inflammatory and anti‐fibrotic phenotype remain to be fully elucidated. Although our transcriptomic data point to relevant pathways, deeper mechanistic insights are needed.

## 4 Conclusion

In conclusion, we have developed and optimized a non‐invasive therapeutic strategy, which employs low‐intensity pulsed US and diagnostic MBs Sonazoid to deliver favorable rhythmic mechanical stimuli to the renal microvasculature. In a murine model of progressive DKD, this US‐triggered MB cavitation protocol significantly improved renal function and structure, enhanced cortical microcirculatory perfusion, and induced a transcriptomic shift toward an anti‐inflammatory and anti‐fibrotic phenotype. These findings demonstrate that the US therapeutic protocol effectively retards DKD progression by restoring microvascular homeostasis and suppressing pathogenic cascades, thereby offering a promising and translational platform technology for the management of DKD and potentially other microvascular diseases.

## 5 Methods

### 5.1 Cell Culture

Primary HUVECs were purchased from the China Center for Type Culture Collection (CCTCC, Wuhan, China) with the catalog number GPC0114. Cells were maintained in a primary EC‐specific medium (CCTCC, Wuhan, China) at 37°C in a humidified incubator with 5% CO_2_. Subsequent experiments were performed when primary HUVECs were passaged to the 10th generation.

### 5.2 In Vitro Intervention of HUVECs

To model the vascular microenvironment, a custom device was designed for exposing cultured HUVECs to US‐triggered cavitation. Culture dishes seeded with HUVECs were completely submerged in medium within the container. Each dish was inverted 180° and secured to eliminate air bubbles. A total of 0.1 mL of Sonazoid (GE Healthcare, Oslo, Norway), diluted 1:100 from stock, was injected into each dish. After a short incubation allowing MBs to rise and adhere to the cell monolayer, cells were exposed to LIPUS at varying MI. Cells were harvested 3 h post‐irradiation for transcriptomic analysis.

To recapitulate the diabetic endothelial phenotype, HUVECs were cultured in high‐glucose Dulbecco's Modified Eagle Medium (DMEM, 30 mM glucose, Gibco) for 2 h. For parameter screening, US frequency was fixed at 3.4‐4.1 MHz, while MI was tested at four gradients (0.2, 0.3, 0.4, and 0.5).

### 5.3 CCK‐8 Assay for Cell Viability

Cell viability was assessed via the CCK‐8 assay (Cat. No. C0037, Beyotime, Shanghai, China) in accordance with the manufacturer's instructions. Cells receiving different treatments were evenly seeded into 96‐well plates. After 24 h of incubation, 10% CCK‐8 solution was added to each well. The plates were further incubated for 1 h. The absorbance value at 450 nm was determined with a microplate reader. Cell viability was calculated by normalizing the absorbance of each experimental group to that of the control group.

### 5.4 Animals

8‐week‐old male mice of two strains were used: C57BLKS/J db/db mice as the DKD model and C57BL/6 mice as the wild‐type control. All mice were obtained from the Model Animal Research Center of Cavens (Changzhou, China). Mice were housed under specific pathogen‐free conditions (21–25°C, 50%–60% humidity, 12/12 h light/dark cycle) with free access to food and water. All animal procedures were approved by the Animal Ethics Committee of the Fourth Military Medical University (Approval No. 20250124) and conducted in compliance with national guidelines.

### 5.5 In Vivo Intervention in DKD Mice

Db/db mice received a standardized dose of Sonazoid via tail vein injection, immediately followed by LIPUS exposure to the renal region for 10 min per session. A non‐focused ultrasound transducer (Sonoscape convex array transducer 3C/A) was positioned on the abdominal surface. Throughout the 10 min treatment, the transducer was continuously manually translated in a craniocaudal direction to ensure complete and uniform insonation of both kidneys. For initial parameter optimization, mice were stratified into four groups based on frequency and MI: (1) 3.4–4.1 MHz, MI = 0.2; (2) 3.4–4.1 MHz, MI = 0.3; (3) 4.0–5.5 MHz, MI = 0.2; (4) 4.0–5.5 MHz, MI = 0.3. The finalized and optimal ultrasonic parameters applied in the formal US therapy protocol were set as follows: US frequency of 3.4–4.1 MHz, mechanical index of 0.2, peak negative pressure of 0.37–0.41 MPa, pulse repetition frequency of 6000 Hz, pulse duration of 0.8 µs, and a duty cycle of 0.48%.

### 5.6 Transcriptome Sequencing

Total renal tissue RNA isolation, RNA sequencing, and subsequent data analysis were all performed following the standard protocols of Novogene Bioinformatics Technology Co., Ltd. (Beijing, China). DEGs were identified with the thresholds of adjusted P value (p‐adj) < 0.05 and |log_2_ Fold Change|≥1. GO pathway enrichment analysis and GSEA were conducted using https://www.bioinformatics.com.cn, an online platform for data analysis and visualization.

### 5.7 Real‐Time Quantitative Polymerase Chain Reaction (RT‐qPCR)

Total RNA was extracted from cells at different experimental stages using an RNA Isolation Mini Kit following the manufacturer's protocol. Then, 1 µg of total RNA was reverse‐transcribed into complementary DNA using HiScript II Q RT SuperMix (Vazyme Biotech Co., Ltd., Nanjing, China) according to the kit instructions. Quantitative PCR was performed using SYBR Green qPCR Master Mix (Vazyme Biotech Co., Ltd., Nanjing, China) on a Thermo ABI QuantStudio 6 Real‐Time PCR System (Thermo Fisher Scientific, Waltham, MA, USA). The primer sequences used in this study are listed in Table S1.

### 5.8 Renal Function Assessment

Renal function was evaluated by measuring serum creatinine, serum urea, serum uric acid, UACR, and GFR. Serum creatinine, urea, and uric acid levels were detected using a Rayto Chemray 800 Automatic Biochemical Analyzer (Rayto Life and Analytical Sciences Co., Ltd., Shenzhen, China) with the corresponding Rayto assay kits: creatinine assay kit (Catalog No. S03076), urea assay kit (Catalog No. S03036), and uric acid assay kit (Catalog No. S03035).

Urine samples were collected using self‐made metabolic cages over a 24 h period. Urine microalbumin concentration was measured using a urine microalbumin kit (Catalog No. ml063626, ML Bio Technology Co., Ltd., Shanghai, China), and urine creatinine concentration was determined using a creatinine assay kit (Catalog No. C011‐2‐1, Nanjing Jiancheng Bioengineering Institute, Nanjing, China). UACR was calculated as the ratio of urine albumin to urine creatinine.

GFR was assessed by plasma clearance of fluorescein isothiocyanate (FITC)‐labeled inulin. FITC‐inulin (Cat. No. F3272, Sigma‐Aldrich, St. Louis, MO, USA) was dissolved in sterile PBS (10 mg/mL) and filtered through a 0.20 µm syringe filter (Sartorius AG, Göttingen, Germany). FITC‐inulin (100–200 µL) was administered via the tail vein; syringe weight before/after injection confirmed the exact dose. Serial blood samples were collected from tail tips at 1, 3, 5, 10, 15, 35, 55, and 75 min post‐injection into pre‐heparinized tubes. Plasma was isolated by centrifugation (3000 rpm, 10 min, 4°C) and kept light‐protected. Diluted in PBS with 500 mM HEPES (pH 7.4), plasma fluorescence (excitation/emission: 485/538 nm) was detected using a SpectraMax iD3 microplate reader (Molecular Devices, San Jose, CA, USA) in black 96‐well plates. FITC‐inulin clearance was calculated via non‐compartmental pharmacokinetic analysis, defined as the intravenous FITC‐inulin dose divided by the total area under the plasma fluorescence‐time curve (AUC_0__−_∞) [35].

### 5.9 Histological, Immunohistochemical, and Immunofluorescence Staining

Kidney and liver tissues were fixed in 4% paraformaldehyde (pH 7.4) overnight at 4°C, embedded in paraffin, and sectioned into 3.5 µm‐thick slices. For general histology, sections were subjected to hematoxylin and eosin (H&E), Masson's trichrome, and periodic acid‐Schiff (PAS) staining. For immunohistochemistry, antigen retrieval was performed, followed by blocking and incubation with an anti‐F4/80 antibody (1:400, Cell Signaling, 70076s) to detect macrophages. For immunofluorescence, sections were processed similarly and incubated with an anti‐NPHS2 (1:400, Abcam, ab181143) antibody to label podocytes. After applying appropriate secondary antibodies (HRP‐conjugated for IHC or fluorophore‐conjugated for IF) and counterstaining, all sections were imaged using a light microscope (SWe‐CX63, Servicebio) or a fluorescence microscope (Eclipse C1, Nikon). Quantitative analyses were performed using ImageJ.

### 5.10 Transmission Electron Microscopy

Kidney tissue ultrastructure was observed using a transmission electron microscope (HT7800, Hitachi High‐Technologies Corporation, Tokyo, Japan) following conventional protocols. Briefly, kidney tissue samples (1 mm^3^) were fixed in 2.5% glutaraldehyde in 0.1 mol/L phosphate buffer (pH 7.4) at 4°C overnight, followed by post‐fixation in 1% osmium tetroxide (OsO_4_) for 2 h at room temperature. After gradient dehydration with ethanol and acetone, the samples were embedded in epoxy resin. Ultrathin sections (60‐80 nm) were cut, stained with uranyl acetate and lead citrate, and observed under the TEM. Digital images were acquired for analysis.

### 5.11 Super‐Resolution Ultrasound Localization Microscopy

Mice were anesthetized with isoflurane gas (induction: 3%–5%, maintenance: 1%–2%) and fixed in a supine position. B‐mode images of the short‐axis section of the mouse kidney were acquired using a VINNO Ultima9E ultrasound system (VINNO Technology Co., Ltd., Jiangsu, China) equipped with an X10‐23L US probe. After fixing the probe, 100 µL Sonazoid (GE Healthcare, Oslo, Norway) was injected via the tail vein, and 10 s ULM images were acquired immediately at a frequency of 13 MHz, an MI of 0.6, and a calibrated peak negative pressure of 2.16 MPa. Using the VINNO ultrasound workstation, three renal cortex regions of interest (ROIs) were delineated per sample. Microvascular parameters, including mean vessel density, vessel ratio, vessel velocity, and perfusion index, were analyzed.

### 5.12 Statistical Analysis and Reproducibility

All data were analyzed and plotted using GraphPad Prism 9.5 and Adobe Illustrator 2024. Data are presented as the mean ± standard error of the mean (SEM). Unless otherwise stated, all experiments were performed with at least three biological replicates. Comparisons between two groups were analyzed using two‐tailed t‐tests. For comparisons among more than two groups, one‐way analysis of variance (ANOVA) was used followed by Tukey's post hoc test. Statistical significance was defined as ^*^ *p* < 0.05, ^**^ *p* < 0.01, and ^***^ *p* < 0.001. Specifically, in Figure 3, ### denotes p < 0.001 for DKD + Therapy vs. WT.

## Author Contributions

**Yubo Lai**: Conceptualization, Methodology, Investigation, Formal analysis, Writing – Original Draft. **Liang Zhang**: Investigation, Validation, Data Curation. **Wenxin Tao**: Investigation, Validation, Data Curation. **Jieyuan An**: Methodology, Visualization. **Lantian Wang**: Methodology. **Zhaoyou Liu**: Visualization. **Guodong Yang**: Supervision, Project administration. **Pengying Wu**: Conceptualization, Supervision, Funding acquisition, Writing‐ Review & Editing. **Lijun Yuan**: Conceptualization, Supervision, Funding acquisition, Writing – Review & Editing.

## Ethics Statement

All animal experiments were performed in accordance with the National Institutes of Health Guide for the Care and Use of Laboratory Animals and were approved by the Animal Ethics Committee of the Fourth Military Medical University (Approval No. 20250124). All efforts were made to minimize animal suffering and to use the minimum number of animals necessary for statistical reliability.

## Conflicts of Interest

The authors declare no conflicts of interest.

## Supporting information




**Supporting File**: advs77989‐sup‐0001‐SuppMat1.docx.

## Data Availability

The data that support the findings of this study are available in the Supporting Information of this article.
