# Supplementary material for: Low‐Intensity Ultrasound‐Triggered Microbubble Cavitation Attenuates Diabetic Kidney Injury Partially by Reversing Endothelial Dysfunction
Source: Adv Sci (Weinh). 2026 Oct 6:e77989. Online ahead of print. doi: 10.1002/advs.77989 (PMC13643233; doi:10.1002/advs.77989)
Supplement: Supplementary file 1 [file ADVS-9999-e77989-s001.docx]

**Low-Intensity Ultrasound-Triggered Microbubble Cavitation Attenuates Diabetic Kidney Injury Partially by Reversing Endothelial Dysfunction**

*Yubo Lai^1^*, Liang Zhang^1^*, Wenxin Tao^1^*, Jieyuan An^1^, Lantian Wang^1^, Zhaoyou Liu^1^, Guodong Yang^2^, Pengying Wu^1^*, Lijun Yuan^1^**

1 Department of Ultrasound Medicine, Tangdu Hospital, Fourth Military Medical University, Shaanxi 710038, China.

2 State Key Laboratory of Holistic Integrative Management of Gastrointestinal Cancers, Department of Biochemistry and Molecular Biology, Fourth Military Medical University, Xi’an, Shaanxi 710032, China.
Corresponding authors: *Pengying Wu^1^, Lijun Yuan^1^*

*Pengying Wu*, [wutdyy@163.com](mailto:wutdyy@163.com)

*Lijun Yuan*, [yuanlj@fmmu.edu.cn](mailto:yuanlj@fmmu.edu.cn)

**Figure S1. Quantitative and clustered analysis results for HUVECs transcriptome sequencing.** (A) Correlation analysis of RNA-seq results; (B) Venn diagram of co-expressed genes in HUVECs; (C) Clustered heatmap of DEGs from HUVECs transcriptome sequencing. Clustered heatmap of DEGs from HUVECs transcriptome sequencing.

**Figure S2. GO enrichment analysis results from HUVECs transcriptome sequencing.** (A) DEG enrichment results: MI = 0.2 group vs. control group; (B) DEG enrichment results: MI = 0.3 group vs. control group.

**Figure S3. GSEA enrichment analysis results of DO gene sets associated with DEGs (MI = 0.2 group vs. control group).** (A) GSEA enrichment plot for DO gene set: CHRONIC KIDNEY FAILURE; (B) GSEA enrichment plot for DO gene set: END STAGE RENAL FAILURE;

**Figure S4. Screening of optimal microbubble doses for in vivo US-MBs modulation, detected by RT-qPCR.** (A-F) Relative mRNA expression levels of *Il1b, Il6, Il8, Tnfa, Tgfb1,* and *Ccl2* in renal tissues during MBs dose screening. n = 3, data are presented as mean ± SEM. Statistical significance was determined using one-way ANOVA. n.s. no significance, **P* < 0.05, ***P* < 0.01, ****P* < 0.001.

**Figure S5. Safety evaluation of US-MBs modulation via H&E staining of the liver, kidney, spleen, lung and heart after a 4-week intervention period.**

**Figure S6. Safety evaluation of repeated US-MBs modulation by macrophage immunohistochemistry after a 4-week intervention period.** (A) Representative F4/80 immunohistochemical staining of the liver and spleen after a 4-week intervention period. (B) Quantitative analysis of F4/80-positive area in the liver. (C) Quantitative analysis of F4/80-positive area in the spleen. Data are presented as mean ± SEM (n = 6). n.s., not significant.

**Figure S7. Safety evaluation of US-MBs modulation via serum assays after a 4-week intervention period.** (A-C) Serum levels of the pro-inflammatory cytokines TNF-α, IL-1β, and IL-6, respectively. The gray shaded region indicates values below the sensitivity threshold of the assay kit. (D-G) Serum liver function parameters: albumin (ALB), total bilirubin (T-BIL), aspartate aminotransferase (AST), and alanine aminotransferase (ALT). Data are shown as mean ± SEM. n = 5. ns, not significant.

**Figure S8. Ultrasound exposure field verification and Sonazoid microbubble stability analysis under ultrasound irradiation.** (A) Representative renal B-mode, color Doppler flow imaging (CDFI), and contrast-enhanced ultrasound (CEUS) images of mice in the control group and US treatment group. CEUS images were acquired at 0 min (before contrast agent injection), as well as at 5 min and 10 min after intravenous injection of Sonazoid. (B) Quantitative analysis of relative microbubble signal intensity over the ultrasound exposure period.

**Figure S9. Improvements in renal function and glomerular ultrastructure induced by US-MB-mediated mechanical modulation.** (A) Schematic illustration of the experimental timeline for evaluating sustained therapeutic efficacy. (B) Body weight.(C) Fasting blood glucose (FBG). (D) Serum urea levels. (E) Serum creatinine (CREA) levels. (F) Serum uric acid (UA) levels. (G) Representative transmission electron microscopy (TEM) images of the glomerular filtration barrier. Blue arrows indicate the glomerular basement membrane; red arrows denote podocyte foot processes.(H) Quantitative analysis of glomerular basement membrane thickness. (I) Quantitative analysis of podocyte foot process width. Data are presented as mean ± SEM (n = 6). Statistical comparisons were performed using one-way ANOVA. n.s., not significant; **P* < 0.05, ***P* < 0.01, ****P* < 0.001.

**Figure S10. Ultrasound-only intervention fails to ameliorate renal inflammation and improve renal function in DKD mice after a 4-week intervention period.** (A) Quantitative analysis of urine albumin-creatinine ratio. (B) Quantitative analysis of glomerular filtration rate. (C-H) Relative expression levels of *Il1b*, *Il6*, *Il8*, *Tnfa*, *Tgfb1* and *Ccl2* in renal tissues following single ultrasound intervention. n.s., not significant.

**Figure S11. NPHS2 immunofluorescence staining.** (A) Representative images of NPHS2 immunofluorescence staining. Scale bar, 50 μm. (B) Quantitative analysis of the NPHS2-positive area ratio. n = 6. NPHS2 (podocin) is a core slit diaphragm protein specifically expressed in podocytes. ****P* < 0.001.

**Figure S12. Supplementary images of ULM imaging results.** (A) 2D ultrasound images and ULM angle images. (B) Quantification of maximum density index; (C) Quantification of minimum density index. (D) Standard deviation (SD) of the density index. (E) Quantification of complexity level. (F) Quantification of maximum velocity. (G) Quantification of minimum velocity. (H) SD of velocity. (I) Quantification of mean curvature. **P* < 0.05.

**Figure S13. Supplementary results of transcriptome sequencing in mouse renal tissues.** (A) Pearson correlation analysis between samples. (B) GSEA enrichment plots: Downward enrichment trends of the upregulated gene subset derived from the endothelial cell upregulated DEG set (single-cell sequencing). (C) GSEA enrichment analysis of inflammation-related GO gene sets.

**Table. S1 Primers used in this study**

| **Primer** | **Forward (5'-3')** | **Reverse (5'-3')** |
| --- | --- | --- |
| *Tnfa* | CCCTCACACTCAGATCATCTTCT | GCTACGACGTGGGCTACAG |
| *Tgfb1* | GCAACAACGCCATCTATGAGAAA | AACGCCAGGAATTGTTGCTATAT |
| *Ccl2* | TCACCAGCAAGATGATCCCA | CTTGAGCTTGGTGACAAAAACTA |
| *Il1b* | CAGCACATCAACAAGAGCTTCAG | GAGGATGGGCTCTTCTTCAAAGA |
| *Il6* | TGGAGTACCATAGCTACCTGGA | TGGAAATTGGGGTAGGAAGGAC |
| *Il8* | CAAGGCTGGTCCATGCTCC | TGCTATCACTTCCTTTCTGTTGC |
| *Actb* | GTGACGTTGACATCCGTAAAGA | GCCGGACTCATCGTACTCC |
